# Enlightening Butterfly Conservation Efforts: The Importance of Natural Lighting for Butterfly Behavioral Ecology and Conservation

**DOI:** 10.3390/insects9010022

**Published:** 2018-02-12

**Authors:** Brett M Seymoure

**Affiliations:** Department of Biology and Department of Fish, Wildlife, and Conservation Biology, Colorado State University, Fort Collins, CO 80523, USA; brett.seymoure@gmail.com; Tel.: +1-970-495-2004

**Keywords:** anthropogenic factors, light pollution, polarized light, sensory pollution

## Abstract

Light is arguably the most important abiotic factor for living organisms. Organisms evolved under specific lighting conditions and their behavior, physiology, and ecology are inexorably linked to light. Understanding light effects on biology could not be more important as present anthropogenic effects are greatly changing the light environments in which animals exist. The two biggest anthropogenic contributors changing light environments are: (1) anthropogenic lighting at night (i.e., light pollution); and (2) deforestation and the built environment. I highlight light importance for butterfly behavior, physiology, and ecology and stress the importance of including light as a conservation factor for conserving butterfly biodiversity. This review focuses on four parts: (1) Introducing the nature and extent of light. (2) Visual and non-visual light reception in butterflies. (3) Implications of unnatural lighting for butterflies across several different behavioral and ecological contexts. (4). Future directions for quantifying the threat of unnatural lighting on butterflies and simple approaches to mitigate unnatural light impacts on butterflies. I urge future research to include light as a factor and end with the hopeful thought that controlling many unnatural light conditions is simply done by flipping a switch.

## 1. Introduction

Over the last two hundred years, humans have dramatically changed the lighting conditions on Earth [1,2]. This change in lighting includes anthropogenic lights during the night, anthropogenic fires and gas flares, as well as the destruction of habitats that produced distinct light environments [3,4,5]. In fact, nearly all protected areas across the world have had an increase in night time lighting since 1992 [6,7], and it is estimated that over eighty percent of humans live under light polluted skies [2]. Furthermore, 65% of tropical Asian and Sub-Saharan African forests have been lost, whereas only 10% of Mediterranean forests remain, and 36% of tropical rainforests have been destroyed [8]. Of the remaining forests on our planet, 70% are one kilometer from an edge [9]. Thus, the natural light conditions that forest canopy provide have been greatly reduced by human activities [9]. This alarming change in natural lighting conditions has direct ecological consequences including loss of biodiversity and risk of species extirpation and extinction [10,11,12,13].

Previous research has documented the effects of changes to natural light conditions on wildlife ranging from changes in predation, reproduction, phenology, migration and orientation, community level interactions, behavior, communication, and physiology [10,13,14,15,16,17]. However, our understanding of how changes in natural light conditions affect butterfly behavior and conservation status remains largely unknown. Here, I introduce the problem of unnatural lighting, both diurnal (habitat destruction and change) and nocturnal (anthropogenic lighting), in the context of butterfly ecology and conservation. I then review what is known about the importance of light for butterflies across myriad biological functions ranging from, but not limited to, phenology, orientation, foraging, predator-prey interactions, and reproduction. Lastly, I introduce a framework for furthering our understanding of the effects of unnatural lighting on butterflies and the steps to mitigate unnatural lighting on butterflies. As butterflies are a “charismatic” fauna, focusing on conserving natural light conditions in the context of preserving butterfly biodiversity may be an excellent way to conserve natural light conditions for all species including the less “charismatic” species like bats and moths, which may be more vulnerable.

## 2. Nature and Extent of Light

Natural light conditions are dependent upon time, space, and environmental factors [5,18]. Light conditions change throughout the day, night and year. Lighting is also dependent upon the landscape (e.g., forest vs. savannah), weather, lunar cycle and celestial bodies. Furthermore, lighting is complicated by its own physical properties which include wavelength, frequency, polarization, hue, chroma, and intensity [19]. It is vital that lighting is studied with all of these parameters in mind as biological functions have evolved under specific lighting conditions that depend upon time, space, weather, and the spectral properties of light. 

### 2.1. Physical Parameters of Light

**Figure 1:**
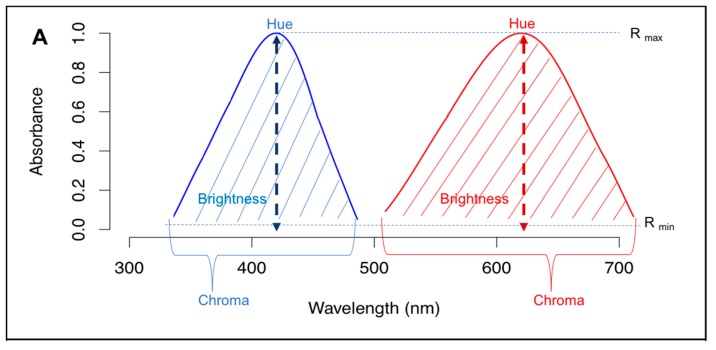

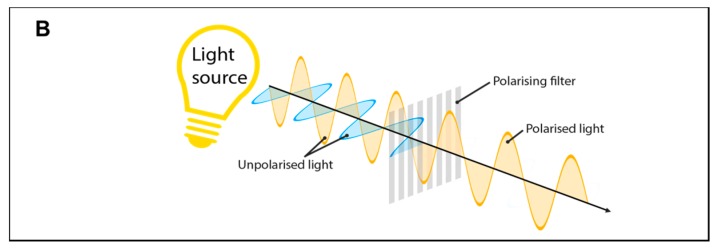
Properties of light. (**A**) Graphic illustration of two different spectra with respective color metrics. The blue spectrum has a peak wavelength of 420 nm whereas the red spectrum has a peak wavelength of 620 nm. Thus, the hue of the blue spectrum is 420 nm and the hue of the red spectrum is 620 nm. The blue spectrum spans a shorter range of the spectrum and thus is more chromatic than the broader red spectrum. The shading under each spectrum represents overall brightness and as the red spectrum is larger than the blue spectrum, it has a greater brightness. For a more in-depth description of color parameters and formulae, see [21]. (**B**) A graphic representation of polarized light. A light source produces unpolarized light, in which the e-vectors of light are oriented randomly, then as the light travels through the filter, only light in one orientation is transmitted resulting in polarized light. Figure 1**B** was adopted from physics.stackexchange.com ©.

What is light? Light is part of the electromagnetic spectrum and can be understood as a stream of photons and a collection of electromagnetic waves, see [19]. Photons have only three properties: frequency, wavelength, and polarization. Wavelength and frequency are inversely proportional and in biology, wavelength is the main property that is used to describe the perceived color of the photon, as most studies focus on eyes absorbing specific wavelengths of photons [19]. Polarization, at its simplest, can be defined as the direction of the wave of light (see [19] for an excellent technical discussion of polarization). However, it is very rare that an isolated photon has biological context and in most cases, we as biologists study spectra comprised of billions of photons. These spectra are histograms of photons of light over a range of wavelengths, usually 300 nm to 700 nm as most organisms have visual abilities within this range [19,20]. Spectra have their own properties and can be described by the parameters: brightness, hue, chroma, and polarization [19], see Box 1 and Figure 1. Briefly, brightness is usually the total amount of photons comprising the spectra and can be measured by taking the integral of the spectral curve [21]. Hue describes the color of the spectra and is usually measured with the peak wavelength [21]. Chroma describes the saturation or ‘peakiness’ of the spectra and is usually measured as a ratio of different bins of the spectrum [21]. For example, a monochromatic red light is highly chromatic whereas pink is less chromatic and white has little chroma (see Figure 1). It is important to note that there are many different metrics for these parameters and for a detailed description of color metrics, see [21]. Lastly, polarization is not calculated from spectra, but instead is measured using polarizing filters or waveplates, see [22,23].

Box 1 **Understanding Light Terms.**

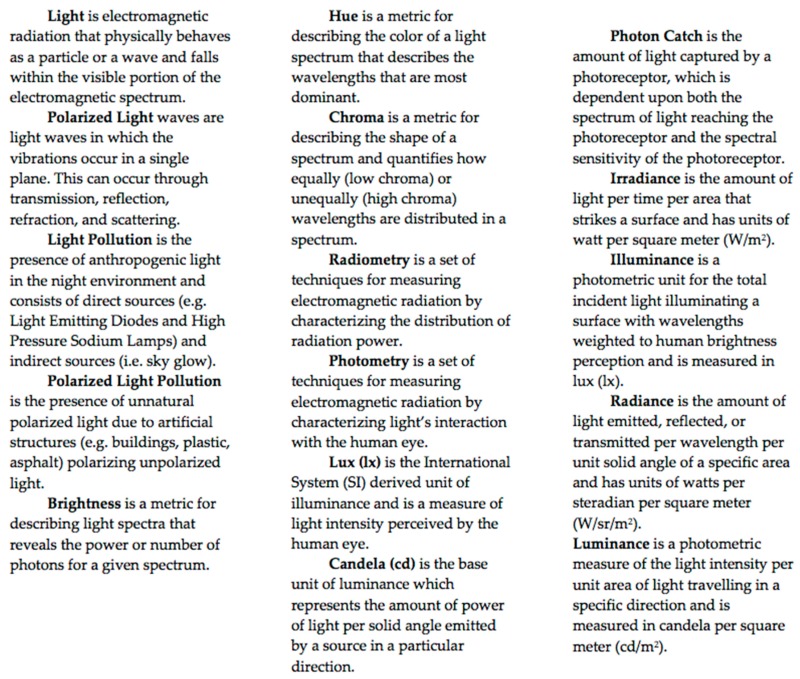

These four properties are very important for biological phenomena and butterflies have been shown to use all of these properties for specific biological functions including reproduction, phenology, mate choice, and foraging, as I will review in the next section. Thus, it is important to realize that light is not created equally, and light of the same intensity can have drastically different effects on animals based solely on wavelength and/or polarization. And perhaps most importantly, we must realize that butterflies, and most animals in fact, have very different visual abilities than humans, and so we may perceive light completely differently from our study organisms (see Light Reception in Butterflies, Section 3).

### 2.2. Natural Light Composition Is a Function of Several Environmental Factors

Biologically meaningful light can be made two ways: thermal radiation (e.g., the sun and electric lighting) and luminescence (e.g., light produced from chemical reactions as exemplified by bioluminescent organisms). As there are no known bioluminescent butterflies, although there are cases of fluorescent *Heliconius* butterflies [24], I will restrict myself to thermal radiation as the sole meaningful type of light for butterflies. The natural thermal radiative light source for diurnal butterflies and for most diurnal terrestrial organisms is the sun and although many nocturnal organisms use light reflected from the moon, the original source is still the sun, although stars have been shown to be important to insects [25]. However, since the invention of electric lighting, the sun will still be the most direct light source during the day, but at night, we have introduced many “suns” and thus animals now have to contend with light at unnatural times and locations. Both the sun and anthropogenic lighting have unique characteristics that are important to biological functions and deserve special attention.

**Figure 2:**
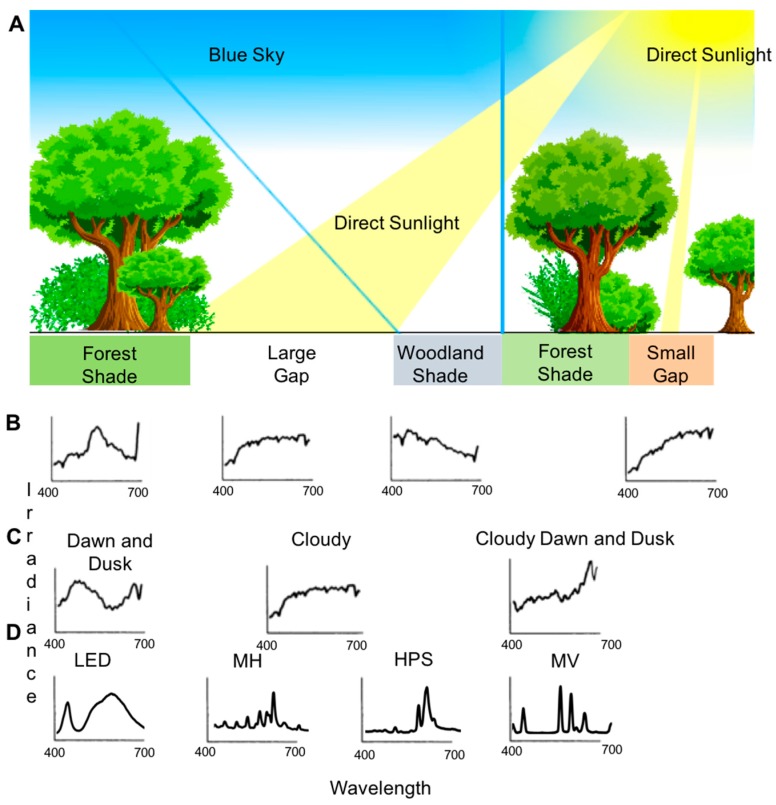
Natural light environments, their spectra, and anthropogenic light spectra (**A**) The four main types of distinct light environments found in forest habitats: forest shade, small gap, woodland shade, and large gap. Each of these light environments arises from the geometry of vegetation, blue sky, and the sun. Modified from [5]. (**B**) The resulting spectra for each of the four light environments with the label above each subfigure. (**C**) Three natural light environments that are due to time of the day and cloudy conditions. Dawn and dusk lighting is characterized by a ‘purplish’ hue as both short and long wavelengths are dominant. Cloudy conditions make most daily light environments similar to large gaps, with the exception that forest shade will still stay middle wavelength dominant. And lastly, clouds during dawn and dusk will lead to an increased long wavelength spectrum. (**D**) Four selected anthropogenic light at night sources that each have their own distinct spectrum. LED = light emitting diode of 3000 K, MH = metal halide, HPS = high pressure sodium, and MV = mercury vapor. All four anthropogenic light at night sources have unnatural peaks and do not represent any natural light source. For all spectra, wavelengths on the x-axis range from 400 nm to 700 nm to stay consistent with previous research [5], and the y-axis is normalized irradiance in photon flux. Thus, these spectra do not represent differences in intensity, only in spectral shape.

Until recently on a geologic timescale, the only sources of light on Earth were the sun, natural fires, and bioluminescent organisms. Thus, organisms evolved under consistent light regimes of day and night, lunar cycles, and seasonality. These natural light regimes have predictable characteristics including intensity and spectral composition. First, the intensity of natural light environments changes 11 orders of magnitude ranging from 10^6^ lux under direct sunlight to 10^−4^ lux during night with cloudy new moon conditions [26]. Furthermore, the spectral composition changes throughout the day, most noticeably during twilight hours when the sun is low on the horizon and much light is scattered through the atmosphere, Figure 2. During daytime, the ambient lighting is bright and rich in most wavelengths of light from below 400 nm to past 700 nm. The shorter wavelengths of light, although originally produced from the sun, come from the sky due to the atmosphere scattering shorter wavelengths of light. The middle and longer wavelengths of light are a result of direct sunlight. However, as the Earth rotates setting the sun below the horizon, the ambient lighting becomes “bluer” with short wavelength light dominating the spectra [5,19,27]. Then as night begins, the spectral composition depends on celestial bodies as the moon reflects sunlight and thus delivers a similar spectral composition to that of the day, albeit 6 orders of magnitude dimmer [19]. However, if the moon is absent, then the ambient lighting will be a result of starlight, atmospheric diffuse light, and airglow and thus middle wavelength rich [28,29]. At such low light intensities, few animals are able to perceive color and no butterfly is known to be able to perceive color at such low light levels. As far as a butterfly is concerned, the more important spectral differences in natural lighting are due to the geometry of other daily environmental factors such as clouds and vegetation. 

The ambient lighting that a diurnally active butterfly will experience is dependent upon the geometry of the sun, clouds and other weather, and vegetation. If a butterfly is flying in an open field or above a forest canopy (termed large gap [5], Figure 2), then vegetation does not come into play and the butterfly will experience bright full spectrum light unless clouds block the sun. However, if a butterfly is flying through a forest, there are several different light environments available due to forest vegetation: woodland shade, forest shade, and small gap [5], Figure 2. Imagine a butterfly flying at the edge of the forest with the sun blocked by the canopy but the blue sky is visible for half of the hemisphere, which will result in the longer wavelengths of light being blocked by trees and shorter wavelengths of light (i.e., blue) dominating the light environment. As the butterfly turns into the forest, the blue hemisphere will also become blocked and the light environment will become much dimmer, by about an order of magnitude, and the light will be rich in middle wavelengths due to most light being filtered through chlorophyll in leaves. Then as the butterfly continues to fly around the forest, it will most likely reach small sun flecks, in which there is a hole in the canopy and direct sunlight reaches the forest floor resulting in a bright, longer wavelength rich light environment [5,30], Figure 2. Lastly, clouds and solar elevation can drastically change the light environment that an organism experiences, and the general trends are that clouds will reduce the spectral hue of forest environments resulting in homogenous full spectrum light, whereas low solar elevation will result in a purplish hue (middle wavelengths absent). Thus, butterflies can experience numerous natural light conditions throughout their day including large gaps, small gaps, woodland shade, forest shade, dawn and dusk, as well as different gradations of these environments due to clouds (for an extensive appraisal on diurnal light environments, see [5]). And although research is limited, there is growing evidence that butterflies can cue into these different environments for numerous biological functions as will be discussed in the next section.

### 2.3. Anthropogenic Lighting and the Built Environment Produce Unnatural Light Conditions

**Figure 3:**
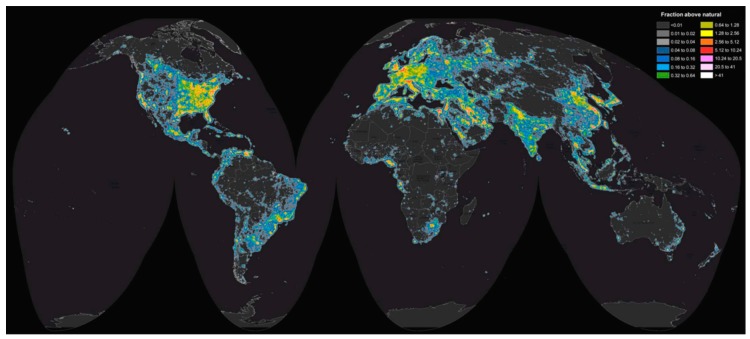
World map of artificial sky brightness. The map shows, in twofold increasing steps, the artificial sky brightness as a ratio to the natural sky brightness (assumed to be 174 μcd/m^2^). The colors represent the amount of artificial brightness with warmer colors indicating higher levels of artificial brightness. For details on the study, please see the manuscript: Falchi et al. (2016), the new world atlas of artificial night sky brightness [2]. Reprinted from *Science Advances*, Falchi et al. (2016). © The Authors, some rights reserved; exclusive licensee American Association for the Advacement of Science. Distributed under a Creative Commons Attribution NonCommercial License 4.0 (CC BY-NC) http://creativecommons.org/licenses/by-nc/4.0/.

The natural patterns of light have become drastically altered by the built environment and the invention of anthropogenic light. The global spread of anthropogenic light has been poignantly demonstrated with the use of satellite data, see Figure 3. This anthropogenic light has been introduced in places, times, and with both unnatural intensities and unnatural spectral composition [31]. Anthropogenic light, termed artificial light at night (ALAN), comes from myriad sources including street lighting, advertising lighting, architectural lighting, security lighting, domestic lighting, and vehicle lighting [12]. Furthermore, ALAN is not spatially restricted from its source and light can travel hundreds of kilometers through the atmosphere and result in sky glow, which is easily observed when traveling towards an urban center at night [32,33]. The spectral composition can also vary greatly depending on the type of lighting used (e.g., high pressure sodium, mercury vapor, metal halide, LED, etc., see Figure 2B) and current governmental efforts appear to be selecting light sources with “whiter lighting” such as higher color temperature LEDs, which can be rich in shorter wavelengths of light [3]. This transition from longer wavelength light sources (i.e., sodium lamps, see Figure 2B) to shorter wavelength LEDs is alarming due to the known effects of shorter wavelengths of light contributing more to sky glow [33,34]. For specific spectral characteristics of light sources see Figure 2B and for further information of ALAN light sources, see [35]. Lastly, I must be clear that we have two different light problems: (1) the destruction of natural light conditions through altering the natural environment (i.e., deforestation and the built environment); and (2) through lighting the nocturnal environment with anthropogenic light sources. Anthropogenic lighting during the day (although very rare) should have no ecological consequences as direct sunlight will drown out the anthropogenic light.

The light source is not the only player in artificial light at night as other manmade structures can greatly affect the amount of artificial night lighting in an environment. The two main concerns are the fixture in which the light is placed and surrounding surfaces (e.g., sidewalks, buildings, etc.) [36]. Dependent upon the light fixture itself, anthropogenic night light can be illuminating all directions or can be directly illuminating areas through shielding (see Moving Forward, Section 5). It is important to note that anthropogenic night lighting, regardless of light source type, can still be greatly affecting natural nocturnal light environments due to the direction in which light is emitted from the source. Furthermore, manmade structures can greatly enhance or mitigate anthropogenic light through reflectance or absorbance, respectively. If buildings are blocking light sources from illuminating natural areas, then light pollution to an area will be minimized. However, if buildings are constructed with highly reflective materials (e.g., concrete, glass, etc.) and are illuminated by artificial light sources, light pollution can increase.

Lastly, anthropogenic light is not only a threat through illuminating natural landscapes with greater intensities and different spectra than normal, but anthropogenic light can cause unnatural polarized light conditions, which can greatly confuse, disorient, and, in some cases, lead to the death of organisms [37]. Water is a very common natural light polarizer and many organisms use polarized light as a cue to find water for habitat use, egg laying, and many other important biological functions [37]; however, artificial structures such as plastic sheets (for agricultural use) and asphalt roads in combination with natural and/or artificial lighting can polarize light and lead animals to behave as though they are near water. This can lead to aerial animals crashing into non-water surfaces [38], laying aquatic eggs on dry surfaces [39], and other behaviors that are harmful to an organism’s fitness; see [37] for an extensive review. Little research has been conducted on the importance of polarization in butterfly biology, although it is known that butterflies are able to perceive polarized light and have behaviors dependent upon it as will be discussed further in the next sections.

### 2.4. A Problem with Studying Light—The Units

Before reviewing what is known about the importance of light in the lives of butterflies, it is important to address an issue that keeps many biologists away from incorporating light into their studies, and that is the complicated nature of the units. Humans have been studying light for millennia and thus many different approaches and units have been derived. Unfortunately, many units are based on the human visual system (i.e., photometric units) and therefore are very limited for application to other organisms as most organisms have very different visual systems, as I will enumerate in the next section. To be able to apply light measurements to any context, one needs to measure the spectra (i.e., radiometric units) of light in wavelengths from 300 nm to 800 nm as this is the range of light that organisms are able to perceive. These measurements are histograms of photons (or quanta) for each wavelength (or frequency) per unit time per area. Most spectroradiometers measure light in quanta and thus the units are watts per square meter per nanometer. However, visual systems count photons, not energy, and thus measurements should be converted to photons per second per square meter per nanometer (see [19] for conversions and a more detailed description of units). These units, commonly called photon flux, are the gold standard for measuring biologically meaningful light.

The measurement of light becomes more complicated as light can be measured as coming from a source (radiance in radiometric units, luminance in photometric units) or as hitting a surface (irradiance in radiometric units, illuminance in photometric units). And because radiance is measuring the light leaving a source in space, it must have a solid angle measurement included. As irradiance is the light hitting a surface, it only requires the size of the surface area. It is important to acknowledge the difference. To understand the importance of light in the lives of butterflies and other insects, we must as a field approach the study with the same measurements to build a comparative database. Furthermore, these light metrics have different units dependent upon whether a radiometric unit is used or whether a photometric unit is used. Unfortunately, most studies use photometric units as the equipment is usually inexpensive relative to radiometric equipment. The most common photometric unit for irradiance is lux, which is a unit weighted to the visual response of humans. The most common photometric unit for radiance is the candela. These units are important to note as much research uses these instead of the radiometric units and, thus, these studies should be interpreted cautiously as the spectra of lights have been measured through a ‘filter’ of human vision. Moving forward, we must measure light in radiometric units and if a photometric comparison is needed, conversions can easily be conducted; see [19].

## 3. Light Reception in Butterflies

### 3.1. Butterfly Vision

Butterflies, like many arthropods, have two compound eyes, each of which comprises a hemispherical array of thousands of individual photoreceptive elements called ommatidia [40]. Ommatidia are comprised of a facet lens at the distal end, which focuses light through another lens, the crystalline cone, which focuses light onto the rhabdom. The rhabdom has up to nine photosensitive cells that will absorb specific wavelengths of light, resulting in color vision. Some species of butterflies have up to five different types photoreceptors that are sensitive to a specific range of the light spectrum [41]. Each of these photoreceptors has an axon that communicates with processing centers outside of the eye in the central nervous system.

Butterflies, unlike most moths, have apposition compound eyes in which light entering the facet is only focused on photoreceptor cells within that ommatidium. Moths, however, have superposition eyes that can focus light entering one facet onto many different photoreceptor cells of neighboring ommatadia enhancing the ability to detect light, an adaptation for seeing in dim light. Sensitive vision depends on many morphological and physiological traits; see [40,42,43,44]. Briefly, sensitive vision in butterflies results from bigger facets, larger eyes, a tapetum that reflects unabsorbed light back through the rhabdom (in non-Papilionid butterflies), longer rhabdoms, and neurological mechanisms that enable the butterflies to spatially and temporally sum input from photoreceptors [42,44,45,46]. Thus, butterflies have less sensitive vision due to their apposition eyes, but they do have better spatial acuity than moths.

Butterflies vary in their ability to resolve objects in their environment and previous research has shown that not only are there differences in acuity dependent upon species, but also dependent upon sex, and eye region (e.g., frontal vs. dorsal) [43,47,48]. The acuity of the butterfly eye has acute zones, which is comprised of larger facets that view very similar areas in space. These acute zones are known to occur in regions of the eye that are used for specific behavioral tasks such as finding mates and hostplants.

Furthermore, butterflies are sensitive to polarized light [49,50,51]. This ability to detect polarized light arises from a highly ordered arrangement of the internal components of a photoreceptive cell, see [40,51] for details. Briefly, if the visual pigments within the photoreceptors are arranged perpendicular to one another, the central nervous system can encode the specific plane of polarization of light [40,50,52]. Previous research has shown that butterflies use their ability to detect polarized light in mate choice and Monarch butterflies (*Danaus plexippus*) utilize the angle of the polarized skylight to orient during migration [52,53,54,55].

### 3.2. Non-Visual Light Reception in Butterflies

In arthropods, extraocular photoreception is common and non-visual photoreceptors have been found in both the central nervous system and in the peripheral nervous system as sensory neurons [56,57,58,59]. Butterflies exhibit a diversity of extraocular photoreception including genital and antennal, which are crucial for reproductive behavior, circadian rhythms, and migration, respectively [54,58,60].

Arikawa et al. (1980) discovered extraocular photoreceptive areas on the genitalia of *Papilio xuthus* and then in follow up work confirmed that 15 other species of butterflies had genital photoreceptors and that the wavelength sensitivity was mostly in the 350 nm to 500 nm range [59]. These genital photoreceptors are important for males to copulate with females as males need light to open their valva, which are needed to clasp onto females during copulation [61]. Females require light stimulation of the genital photoreceptors to oviposit and if these photoreceptors do not detect light, females cannot lay eggs [62,63]. However, the genital photoreceptor research is limited to very few species of butterflies and whether this trait is phylogenetically conserved across Papilionoidea remains uninvestigated. Furthermore, whether the spectral sensitivity of these extraocular photoreceptors varies between species requires further research.

The comprehensive work into the Monarch (*Danaus plexippus*) migration by Reppert and his colleagues has been immensely informative for understanding the role of extraocular photoreceptors for butterfly biology. Reppert et al. (2004) have shown that the spectacular migration of Monarchs, which can be up to 4000 km long, is dependent upon both the timing of the light cycle and the natural polarization of the sky [54,55,60,64]. Briefly, the photoreceptors in the antennae of the Monarch are cryptochromes and not opsins, and it is these cryptochromes that are the light-input pathway for maintaining an internal clock [55,60].

Butterflies have immense light reception abilities including very good color vision, spatial acuity up to several meters, limited dim-light vision, perception of polarized light, and the ability to detect light through non-visual means. Butterflies use light reception for numerous fitness related tasks and as such, any changes in their visual environment through alteration of natural light environments could have drastic consequences. In the next section—ecological implications of unnatural lighting—I will merge concepts of lighting with light reception to enumerate upon the consequences of unnatural light for butterflies in phenology, habitat loss, orientation, reproduction, foraging, predation, and communication.

## 4. Unnatural Lighting Implications for Butterflies

### 4.1. Vulnerable Biological Functions and Underlying Mechanisms

In the last decade, much effort has been taken in investigating the effects of sensory pollution derived from anthropogenic light and noise on organisms across many taxa [10,12,65,66,67]. Most research has focused on one anthropogenic pollutant (i.e., either light or noise), in the context of one biological function (e.g., sleep, reproductive timing, etc.), and in one species [10,67]. Fortunately, the field of anthropogenic effects on sensory ecology has begun to develop frameworks for tackling the myriad hypotheses and predictions relating the effects of anthropogenic light and noise on organisms. I mention the noise pollution work here, not because I believe that noise will affect butterflies, which I do not as most butterflies don’t have tympanic organs [68], but instead because I and other sensory ecologists believe that the framework developed for studying noise pollution will fit well with studying the ecological effects of unnatural light conditions [65,66].

Francis et al. (2013) proposed three mechanisms underlying the effects of noise on organisms: masking, distraction, and misleading. Masking involves an anthropogenic stimulus masking a natural stimulus. Alternatively, an anthropogenic stimulus could distract an organism and alter its natural behavior. Lastly, anthropogenic stimuli can mislead an organism into incorrectly assessing a cue (e.g., a bright mercury vapor lamp as the moon). Although these three mechanisms will work well for predicting the ecological effects of unnatural lighting in many ecological contexts involved with cue and signal assessment, these mechanisms cannot fully incorporate all ecological effects for anthropogenic lighting.

Anthropogenic lighting, unlike anthropogenic noise, also affects organisms due to altering the perceived natural temporal patterns and can either expand or reduce the temporal niche of an organism. If an organism is diurnal, they may extend their activity patterns early in the morning and later in the evening due to greater light levels, whereas a nocturnal animal may reduce their activity patterns due to unnatural light increasing night brightness. Thus, to layout the potential ecological implications for butterflies, I refer to four underlying mechanisms: masking, distraction, misleading, and temporal niche, see Table 1 [65].

Unnatural lighting likely affects many biological functions as well as leading to numerous ecological consequences [10,12,13,69,70,71,72]. I focus on five biological functions: (1) Phenology and Circadian Rhythms; (2) Attraction and Orientation; (3) Foraging; (4) Predation; and (5) Reproduction. I do not include communication as I cover butterfly signaling in predation and reproduction. Furthermore, this list is not exhaustive and other biological functions are likely to be affected by unnatural light conditions. I focus on these five biological functions as there is literature revealing concerns and laying a foundation for direct hypotheses and tests. Lastly, I end this section with discussing how habitat destruction is directly tied to unnatural lighting conditions and possible ramifications for butterflies.

### 4.2. Phenology and Circadian Rhythms

Phenology is the study of the timing of life-history events that are associated with the passage of seasons [109]. Circadian rhythms are timekeeping devices that have an inherent near-24 h periodicity, are protected from changes in temperature, nutrition and pH, and can be tuned to oscillate with a 24 h period (known as entrainment) [110]. Both phenology and circadian rhythms use the natural light/dark cycle (from here on out referred to as photoperiod) of our planet as the environmental input to the physiological system [109]. In this section on phenology and circadian rhythms, I focus on anthropogenic light as this is the most likely to affect phenology and circadian rhythms in butterflies through masking natural environmental cues and/or by misleading butterfly biology through unnatural lighting. Although direct research into the consequences of anthropogenic lighting on the phenology and circadian rhythms of butterflies is lacking, I target three areas in which research exists and highlight needed future research: (1) phenological changes of host plants and nectar sources as bottom up effects and an arms race between hostplants and butterflies (misleading); (2) changes in photoperiodicity due to anthropogenic lighting affect diapause, eclosion and pupation (masking and misleading); (3) changes in lighting affects circadian rhythms in migrating butterflies (masking and misleading).

In herbivorous insects such as butterfly larvae, temporal matching with host plants is widespread [76,77,78,79]. This temporal matching in both plant and insect larvae development sets a stage for an arms race between the two players [79], with the plants needing to develop tannins and other defenses in their leaves before the larvae devour their leaves and reproductive organs (e.g., flowers), and the larvae needing to consume these plant parts before the defenses occur impacting their own survival. Numerous studies have investigated this developmental race between maturing host plants and their insect herbivore [76,77,79,111,112,113,114], and some have shown that climate change is affecting this race with plants receiving an upper hand due to development occurring early in the season from unnatural temperature changes [79,113,114]. The emphasis in this arms race literature has been on the effects of temperature altering this developmental race; however, recent research has shown that anthropogenic lighting is altering the phenology of plants and trees as well [115]. Trees in unnaturally brighter areas budded seven days sooner than plants in areas without anthropogenic lighting [115] and this is likely across many different species of plants [10,13,70]. Thus, anthropogenic lighting is likely affecting the timing of both plant development and larvae development in unequal ways, which could lead to major disruptions in the coevolution between host plants and their insect larvae. This research is needed and simple common garden experiments with host plants and insect larvae under different lighting would greatly increase our understanding of the effects of not just anthropogenic lighting, but lighting effects on an arms race between host plants and insect herbivores that has been developing for millions of years.

Photoperiodism, in which seasonal changes in day length or night length are responsible for directing metabolism, metamorphosis and other physiological processes, has been shown to directly affect the seasonal and daily timing of diapause and eclosion of Lepidoptera, respectively [97,98,116]. To my knowledge, no published studies have demonstrated direct effects of anthropogenic lighting on diapause and eclosion in butterflies. Laboratory studies have shown individual burnet moths (*Pseudopidorus fasciata*) will alter eclosion times under continuous lighting [97], and that diapause in the tobacco hornworm (*Manduca sexta*) is regulated by day length [117]. Although both of these findings are in moths, it is likely that most Lepidoptera, including butterflies, are affected by anthropogenic lighting for both diapause and eclosion. Gotthard (1999) and Sencio (2017) have shown that several different species of butterflies do use the natural light cycle as a cue for diapause and eclosion. Thus, it is likely that lighting is incredibly important for natural metamorphosis in butterflies. Now that anthropogenic lighting is changing day lengths with night being shorter regardless of the season [2,118], it is very likely that butterflies will have unnatural timing of diapause and eclosion under anthropogenic lighting [116]. Simply designed experiments with lights of different intensities and spectra could inform the field on how anthropogenic light will affect both diapause and eclosion in butterflies.

Research into the effects of lighting as the environmental cue in circadian rhythms of insects is amassing and we now have an understanding of the molecular mechanisms of daily rhythms [117,119,120]. The basic tenet is that light is captured by Cryptochrome, a blue light sensitive photopigment, that leads to an enzymatic cascade that sets the internal clock of insects [110]. However, it must be noted that most of our understanding comes from model insects (i.e., *Drosophila*) and very few studies have researched circadian rhythms in butterflies. It is very likely that butterflies use Cryptochrome as the photpigment to entrain the circadian rhythm, but future research into the molecular mechanisms of circadian rhythms in butterflies is needed. We do know from research into the phenomenal North American annual migrations of Monarchs (*Danaus plexippus*), that sunlight, specifically polarized light, is detected by the antennae to aid in a time-compensated sun compass that enables the correct orientation during migration [54,55,60]. Thus, it is probable that butterflies are using natural changes in daily and seasonal light cycles as an environmental cue to drive many biological phenomena. Unfortunately, we currently do not have much research into this mechanism and future work in this realm will greatly inform butterfly conservation efforts with regard to anthropogenic lighting.

### 4.3. Attraction and Orientation

Although scientists and natural historians have been curious of the behavior of butterflies for hundreds of years, we still lack a basic understanding of butterfly attraction to light and their use of light for orientation. There are many anecdotal reports of butterflies avoiding or preferring certain light conditions, but few empirical tests. Unfortunately, most of the empirical tests do not elucidate whether a preference for habitat type or light conditions exist, but instead test light conditions and habitat as they are one and the same [121,122]. This caveat is very important to note, as butterflies may be using light as a cue for locating specific habitats, may be selecting light conditions regardless of habitat type, or butterflies may be cueing into other environmental factors that correlate with lighting conditions. Anthropogenic light sources are likely to be distracting and misleading butterflies from normal behaviors. Research is needed to ascertain the importance of lighting for behavioral attraction and orientation. Here I review previous studies documenting attraction of butterflies to light (both natural and artificial) and the use of light for orientation by butterflies.

The attraction of butterflies to light has been studied in both the contexts of natural and artificial lighting [89,90,95]. Several studies on butterflies have elucidated the attraction of butterflies to artificial lighting and a few key studies exist on butterfly attraction to different natural light conditions. Chowdhury & Soren (2011) reviewed the literature of Indian butterflies as well as inventoried their own data to reveal that since 1951, there have been 27 different species of Indian butterflies from all butterfly families except for Riodinidae documented to be attracted to artificial lighting. Outside of India, very few studies exist documenting the attraction of butterflies to light, although Beshkov (1998) reported ten different butterfly species coming to light traps in Bulgaria and Beshkov posits thermoregulatory hypotheses for these findings; however, no empirical data exist to support the hypotheses [91]. These reports are evidence that butterflies are attracted to artificial lights and it is likely that many more cases exist that have not been published.

Within the last decade, two studies have documented preferences for natural light environments, one in the context of habitat segregation between *Heliconius* mimicry rings [95] and another in the context of mate choice and territorial defense in the speckled wood butterfly (*Pararge aegeria*) [96], which is discussed in the *Reproduction* section below. Seymoure demonstrated that four species of *Heliconius* butterflies prefer different light levels dependent upon light intensity [95]. The four *Heliconius* species comprise two mimicry rings and each mimicry ring occupies a different habitat, forest and open savanna [123,124]. Using an enclosure with two different light environments matching open and closed habitat, Seymoure demonstrated that butterflies preferred the lighting of the habitat in which they naturally occur. Future studies need to replicate these methods to determine if other species that are habitat specialists are using natural lighting conditions to locate and remain in suitable habitats. Once more studies investigate this basic biological phenomenon, we will most likely find that many butterflies rely on natural lighting for habitat selection. Thus, due to habitat degradation, conservation efforts should focus on not only conserving areas, but should focus on conserving the habitats so that natural lighting regimes exist.

Many studies have investigated butterfly orientation in the context of the landscape, see [125,126], but few studies have investigated how butterflies use light to orient in their environment. Research into *Parnassius smintheus* demonstrated that individuals would turn away from forest habitat, although whether the butterflies were cuing into light or landscape remains unknown [121]. The best example of light dependent orientation is in the migrating monarch butterflies which have been empirically shown to use the e-vector of polarized light to orient and navigate during the long migration [54,55,60,99]. It is likely that many butterflies use light intensity and polarized light as a cue for orientation, but we lack a firm understanding of mechanisms [37,54,95]. Further research into butterflies that have a fixed attraction/repulsion to light will greatly increase our understanding of how butterflies use light conditions for orientation and other behaviors. In the future, use of micro-data loggers will be ideal for studying how butterflies and other insects orient through their environment dependent upon lighting.

### 4.4. Foraging

A main ecosystem service provided by adult butterflies is pollination through foraging for nectar [127]. Furthermore, as holometabolous insects, butterflies will be foraging for flowers and other nectar sources as adults, but will feed mostly on leaves as larvae. Both butterfly adults and larvae depend on visual cues and signals to locate food sources and research has shown that adult butterflies rely on color vision to discriminate between correct nectar sources, and that the overall intensity contrast of the flowers to the background are imperative for butterflies to land and feed upon flowers [73,74,75]. Thus, butterflies have evolved specific visual physiology and visually-guided behavior to locate appropriate nutritional sources that are dependent upon the perceived coloration of both the food source and background; and through altering natural light environments, this visual signal between food source to butterfly can be masked by the spectrum of the available light [95]. Ultimately, these unnatural ambient light effects from altered habitat structure (i.e., deforestation), could lead to an inability of butterflies to detect and/or initiate feeding upon the correct food source. This hypothesis has not been tested, but needs to be a goal in the near future to understand how vulnerable species of butterflies are affected by altered light environments for foraging.

Butterfly foraging behavior is likely to be affected by anthropogenic lighting in both adults and larvae. *Heliconius* butterflies forage and find host plants under poor light levels [102] and other species of butterflies have shown temporal specialization in foraging behavior [102]. In the satyrine butterfly, *Lethe diana*, males have been shown to forage early in the morning and then court and defend territories in the afternoon [103]. The proximate mechanism for this switch from foraging to reproductive efforts is unknown, but is likely linked to a circadian rhythm, which would be affected by exposure to anthropogenic lighting at night. Of course, this is another conjecture that further empirical research needs to investigate. As for larvae, research into feeding in Lepidoptera has shown that both light levels and temperature are important [104]. Whether exposure to artificial lighting would increase feeding rates in butterfly larvae is currently lacking empirical evidence; however, research into other insects has shown that foraging is affected by artificial lighting [105].

### 4.5. Reproduction

Butterflies depend upon appropriate light conditions for mate detection [85], courtship [84], mating [58,59], and for successful ovipositing [86]. Numerous studies have shown that butterfly mate detection is dependent upon visual signals across a wide range of wavelengths [24,128,129], and that males orient themselves with respect to the visual environment to increase conspicuous sexual signals [87,88]. Furthermore, the astounding fact that swallowtails use genital photoreceptors to confirm copulation [58,59,61,62], is evidence of the importance of light for butterfly reproduction. Unnatural light conditions are likely to mask, mislead, and lead to inappropriate timing (temporal niche) of mate detection, courtship, mating, and ovipositing in butterflies.

Butterflies have evolved sexual signals in specific habitats and contexts and through unnatural lighting, these behaviors are very likely to be altered. For example, Bergman et al. (2007, 2009) revealed that the male speckled wood butterflies, *Pararge aegeria*, fight over sunspot territories on the forest floor and winners gain sole residency of sunspot, whereas losers patrol the forest looking for females. Male residents achieved twice as many matings due to control of sunspot territory. Furthermore, Bergman et al. (2009) found that females were not interested in the sunspot, but instead that males had increased reproductive success because they were better at locating females flying through a sunspot [96]. Then in another species, the empress leilia butterfly, *Asterocampa leilia*, Bergman et al. (2015) revealed that the contrast of the blue sky and desert scrub affected detection ability of females by perched male butterflies. Lastly, unnatural light conditions are also likely to affect reproductive behaviors in butterflies due to the timing that males begin patrolling for females. Mate finding behaviors in several species of moths and butterflies are dependent upon time of day and most likely entrained by light conditions [97,103,130,131]. And several species of butterflies have consistent eclosion times after sunrise that correspond to mate-locating behaviors [98]. Thus, if the visual landscape is altered by habitat alteration and/or anthropogenic lighting, male butterfly reproductive behaviors could be greatly altered both spatially and temporally.

Another concern for butterfly reproduction that is likely unique to invertebrates is that butterflies use polarized light for reproductive behaviors, and unnatural light conditions alter the natural polarized light environment [52,53,132]. Understory *Heliconius* butterflies use polarized wing reflectance for mate choice, whereas open habitat *Heliconius* butterflies do not have polarized wing reflectance [52]. Understory *Heliconius* butterflies are likely not the only species to use polarized light for sexual signals as Douglas et al. (2007) found that many species of tropical understory butterflies have evolved polarized light signals for mating. As human habitats greatly alter the natural polarized light environment, we need to concern ourselves with the importance of polarized light for butterflies [37].

Lastly, it is intellectually stimulating to hypothesize the effects of anthropogenic lighting on copulations in butterflies as they rely on genital photoreceptors to initiate sperm transfer [58,59,61,63]. Arikawa et al. (1982) found that the spectral sensitivity of the genital photoreceptors has a major peak at 380 nm and a smaller peak at 450 nm. This could be concerning in light of current widespread adoption of LED technologies, especially those with high color temperature (i.e., 5000 K), which have spectral peaks similar to those of the genital photoreceptors. Thus, could anthropogenic lighting enable butterflies to reproduce in appropriate spatial and temporal dimensions that could lead to vulnerability via increased predation due to different predator abundance and behavior? Simple observations in laboratory settings with anthropogenic lighting and reproductive behavior with swallowtails could shed light onto what could be a serious butterfly conservation issue.

### 4.6. Predation

Many butterflies rely on visual signals and coloration as a primary defense against potential predators [133,134,135]. In fact, most butterflies rely on crypsis [136,137,138], warning signals to advertise unprofitability [139,140,141,142], or deimatic coloration (e.g., eye spots) to distract and startle predators [143,144,145]. With regard to visual cues and signals between predators and butterfly prey, unnatural light conditions can mask the signal and lead to an unnatural response from the predator, that would likely decrease butterfly survival [72]. Previous research has shown that attack rates on both butterfly adults and larvae are dependent upon microhabitat, which have specific light environments [100,138]. Grenis et al. (2015) found that Lepidoptera larvae are more likely to be attacked if they were placed in edge, woodland shade habitats during the day, than if they were placed in forest shade habitats. Furthermore, Seymoure et al. (2017) revealed that *Heliconius* butterflies have different attack rates in their respective microhabitats.

Visual cues and signals are likely to be masked by both unnatural light conditions during the day and anthropogenic night lighting. Previous research has shown that butterflies rely on luminance contrast for both warning signals and deimatic displays [80,81]. Sandre et al. (2010) showed that avian predators learned to avoid warning signals dependent upon the brightness contrast and not the specific color. Olofsson et al. (2010) revealed that the eyespots of the woodland brown (*Lopinga achine*) were differentially attacked dependent upon light environment, with butterflies in low light intensities with high UV spectra likely having the eyespot attacked, whereas butterflies in bright light conditions without UV were likely to have the head attacked. Both of these studies are poignant examples of reduced butterfly fitness due to the visual signals being masked by unnatural light conditions. Furthermore, warningly colored butterflies have evolved specific warning signals and behaviors to increase survival [82,83]. Pipevine swallowtails (*Battus philenor*) orient themselves to increase signal efficacy while perched and if anthropogenic lighting changes the spectrum of ambient lighting, these butterflies will have reduced signal efficacy ([116], Seymoure in Prep). Furthermore, Douglas (2013) found that warning colors have evolved dependent upon the light environment in which they are found, with butterflies occupying bright, open light environments having colorful warning signals, and butterflies occupying dim, forest environments having bright, non-colorful, contrasting warning signals. It is likely that unnatural lighting conditions during the day and night are greatly altering the efficiency of evolved predator defenses of butterflies.

Butterflies are not only vulnerable to increased predation due to the masking of visual signals, but also due to misleading environmental information from anthropogenic lighting. As stated above, butterfly larvae alter their rate of development dependent upon photoperiod [101,116,146]. Research has shown that photoperiod is lengthened due to anthropogenic lighting [70,107,147], and thus it is probable that butterfly larvae will be misled into altering their rate of development due to anthropogenic lighting. This is very concerning for two reasons: (1) larvae depend on the natural photoperiod cues to develop in time to diapause during the winter or other harsh climatic events [146,148]; and (2) larvae have much higher mortality due to predators when they have faster rates of development [101]. Thus, altering the perceived photoperiod of larvae through anthropogenic lighting could cause major butterfly mortality.

The previous concerns have focused on direct effects on butterflies, but altering natural light conditions during both day and night may not alter the behavior of the butterfly and yet still be important to butterfly fitness as these light changes could alter the behavior of the predator. In fact, several recent studies have shown that insectivorous birds begin to forage and sing earlier as well as being active later into the evening in the context of anthropogenic lighting [16,106,107]. So even if butterflies do not change their behavior, they may be at greater risk of predation due to an increase of predator activity.

Lastly, many species of butterflies are attracted to anthropogenic lighting and previous research has shown increased predation upon Lepidoptera at anthropogenic light sources [92,93,94]. Although I found no evidence in the literature of predation on adult butterflies at artificial light sources, this is likely due to the methods used, which are either focused on grouping prey into large taxa (i.e., Lepidoptera instead of Nymphalidae) [149,150], or are focused specifically on predator behavior and do not qualify prey [151,152,153] (but see [100] for no effect of anthropogenic light on larvae predation). However, it is likely that anthropogenic lighting affects butterfly predator-defenses due to distracting butterflies through a novel stimulus may leave individual butterflies more susceptible to predation. There are simple approaches to studying this in both the field and lab, and studies could quickly determine if butterflies perched at night change their behaviors when artificial lights are turned on, which in turn could make them more vulnerable to predation.

### 4.7. Habitat Loss and Alteration

Humans have greatly altered the planet’s surface and are the largest cause of habitat loss [154,155,156,157]. Habitat destruction and transformation leads to immediate and delayed biodiversity loss [158], and of the 25 endemic hotspots that have the majority of the planet’s biodiversity, none of these hotspots have more than one-third of their pristine habitat remaining [154]. Most importantly for this review, recent research has shown that butterflies are likely to be affected more from habitat loss than other taxa [159]. And, recent studies have used satellite data of light pollution as a surrogate for habitat loss [155]. Thus, through habitat loss, natural lighting and light regimes are drastically altered resulting in two urgent concerns for butterfly diversity: (1) reduction in natural light environments; and (2) increase in exposure to anthropogenic lighting.

Through natural habitat destruction, the natural light environments produced by the habitat vegetation are removed and, in most cases, a light environment similar to the ‘large gap’ (Figure 2) will replace the myriad of light environments (e.g., ‘forest shade’, ‘woodland shade’) that once provided specific stages for biological functions [5,30,160]. As Endler (1993) demonstrated, a mature forest provides several distinct light environments that are crucial for reproduction, communication, and other fitness related behaviors in several taxa [30,95,160,161,162,163]. Furthermore, through habitat fragmentation, habitats are less likely to have natural understory ‘forest shade’ environments, which are likely imperative to many butterfly species, as these forest fragments will be mostly edge habitat of ‘woodland shade’ light environments. Although direct tests are lacking, observations of forest understory butterflies, especially in the Neotropics, are poignant examples that specialist butterflies like those of the brown butterflies (Satyrinae: example genera of *Pierella* and *Haeterini*) are greatly affected by sun exposure and seek out shaded habitats [102]. By removing large patches of mature forest, we are greatly diminishing any chance for sustainable populations of these specialist butterflies that seek out specific forest light environments.

Through natural habitat destruction, more intact fragments of natural habitat will be exposed to anthropogenic lighting. As forests and other natural habitats are cleared for agriculture and human development, anthropogenic lighting usually is one of the first anthropogenic pollutants due to road and property lighting. Thus, the remaining intact swaths of natural habitats will be exposed to direct and indirect (skyglow) sources of anthropogenic lighting. This effect will again greatly affect those butterflies that are adapted to dim forest environments as they will be exposed to brighter conditions both day and night, and although direct tests are lacking, my preliminary research with *Pierella* has shown that these butterflies do not last long in bright open habitat conditions (Seymoure in prep). Light pollution is likely a threat to butterfly communities as light pollution is growing globally including within world protected areas [6] and as anthropogenic lighting is increasing faster than global population rate due to technological advances reducing the cost and energy demands of anthropogenic lighting [118]. Understanding that anthropogenic light is pervasive throughout fragmented habitats is sobering, but we must as butterfly conservationists address the effects of unnatural light conditions on not just butterflies, but Lepidoptera in general, as there are likely grave consequences if we do not act now to preserve natural light conditions in Lepidoptera habitats. 

Through natural habitat destruction, more of the environment will consist of artificial surfaces such as buildings, roads, dykes, and agricultural fields. This built environment not only introduces unnatural diurnal and nocturnal light conditions, but it also can drastically alter natural polarized light conditions [37]. Again, we understand little of the importance of polarized light for butterfly biology but we do know that butterflies use polarized light to orient and migrate [54,55], court conspecifics [52], signal to putative predators [53], forage and locate hostplants [132], and we know that myriad other insects use polarized light for finding suitable habitats [37,38,39,164]. Furthermore, with the unnatural built environment, humans are introducing novel color schemes and materials that produce unnatural glare, that could affect butterfly behavior resulting in reduced fitness [37].

## 5. Moving Forward

In the previous sections, I have highlighted the underlying butterfly biology that points to unnatural lighting, both diurnal and nocturnal, that could threaten butterfly fitness and result in loss of butterfly diversity. We now, as butterfly researchers, have the challenge to further our understanding of the importance of natural lighting and the impacts of anthropogenic lighting on butterfly fitness to guide and inform conservation and management decisions to mitigate the effects of unnatural lighting on butterflies. Fortunately, butterflies are the ‘charismatic megafauna’ of the insect world and can become the umbrella species for insects and other invertebrates, which are also likely impacted from unnatural lighting. In this last section I briefly introduce methods to quantify the threat of unnatural lighting and reduce the impacts on butterflies of the inevitable lighting changes of the future.

### 5.1. Quantifying the Threat

To fully understand the repercussions of unnatural light conditions on butterfly natural history, we must research not only specific species but also use a wide phylogenetic approach to be able to make predictions for entire genera and sub-families of butterflies. With such a large number of butterfly species, we can’t possibly understand the specific effects for each species under specific circumstances. However, we can use ‘model’ butterfly species as surrogates for butterflies that are rare and/or more difficult to study. We should not be concerned with the specific species of butterfly, but instead, the butterfly behavior and how it as affected by unnatural light conditions. Then, we can map these behaviors onto well-developed phylogenies [68] and use this as a guide for management and conservation.

The easiest path forward is for butterfly biologists to continue to do their research and include measurements of light conditions. Whether they are researching chemical signaling in *Pieris,* mimicry in *Heliconius*, migration in *Danaus*, or mate choice in *Battus*—by including measurements of light intensity and spectral composition, we will better understand the ubiquity of lighting importance for butterflies and how unnatural lighting can have detrimental effects. Although light measurements have likely kept lepidopterists timid from including them in their work, I hope this review shows that measurements of light can be included relatively easily and that wonderful references exist [19,165]. Furthermore, although some light equipment can be expensive to obtain, we can use inexpensive astronomical equipment such as Sky Quality Meters (SQMs; Unihedron, Grimsby, Canada) to at least quantify light changes in butterfly habitats. Another inexpensive method is to use lux meters to quantify overall light changes. Ideally, researchers will incorporate spectroradiometric measurements to fully understand the effects of both brightness and spectral composition on butterfly biology, but as these spectrometers cost more than a thousand dollars, it would at least be beneficial for researchers to invest a few hundred dollars into light meters that can quantify overall light changes. A methods paper utilizing non-traditional light equipment to quantify the effects of unnatural lighting is in preparation and will hopefully guide lepidopterists to include light measurements in their work. Lastly, for butterfly field biologists, there are several excellent light pollution maps that have been recently published and can easily be included into field research as these maps have spatial resolution of near 700 m^2^ (Figure 3; [1,2]). Although these maps are not perfect surrogates for understanding anthropogenic light effects on organisms as they are satellite measurements of upwelling radiance, they are a great start for quantifying anthropogenic light changes on butterflies.

### 5.2. Reducing Ecological Impacts of Unnatural Lighting on Butterflies

Reducing the ecological impacts of unnatural light conditions is very easy in certain contexts and very difficult in others. When reducing blue-dominant anthropogenic lighting, it is easy to mitigate ecological impacts by using a more ecologically friendly night lighting option such as amber LEDs instead of blue-dominant lighting. However, when attempting to restore natural light conditions in a deforested area, there is no ‘flip of a switch’ solution, but only correct forest restoration to renovate the natural forest light conditions that many organisms depend upon. As in most conservation efforts, the best approach is to maintain the natural intact habitats. Of course, we will need to restore deforested areas and when doing so, we must keep in mind the natural light conditions that would have been in the original forest. Thus, if an effort is taken to quickly reforest a neotropical habitat, the native trees must be used instead of bamboo or eucalyptus, as these trees will not provide the correct canopy cover or vegetative stratification needed for producing the different forest light environments [5,160]. Restoring natural daily light conditions will require monitoring and may require very strict forest management to introduce gaps (e.g., treefalls) into maturing forest as treefalls naturally occur from older or dead trees being uprooted by wind [165].

Alleviating the ecological consequences of anthropogenic lighting begins with educating people. Only people are contributing to anthropogenic light at night and in most cases, people are naively lighting their property in ways that can have detrimental ecological consequences [12]. Once informed, most people will alter their lighting to reduce ecological consequences while still performing the function that they require. It will be easier to have people mitigate their lighting effects by installing the proper lighting before they have already invested in improper lighting. Thus, we must educate citizens and local governments about responsible lighting immediately. Several excellent manuscripts and handbooks exist [36,166,167,168], so I will only briefly introduce the three main steps for mitigating anthropogenic lighting: time, direction, and color. Anthropogenic lighting should be used when it is needed and several studies show that constant night lighting does not reduce theft nor increase security [169]. Thus, lights should be set on timers or regulated with motion detectors to turn on when people need them. Also, these lights should be directional and not able to propagate light in every direction. As people will need the light illuminating the ground where they are, proper fixtures are needed to direct light downward and in the appropriate direction. Lastly, animals, including humans, have evolved to use the spectral shape of light as an environmental cue for day and night [70] and therefore, night time lighting should avoid shorter wavelengths of light as these can confuse biological rhythms [16,70]. So, in moving forward to reduce ecological impacts from anthropogenic lighting on all organisms, not just butterflies, we must educate people on responsible night lighting, which is as simple as using directional and long wavelength rich light only when it is needed.

## 6. Conclusions

Unnatural lighting is a global issue that is directly affecting butterfly biology and we are only beginning to understand the detrimental impacts. Both habitat destruction and anthropogenic lighting at night are altering the natural light conditions in which butterflies have evolved specific biological functions for millions of years. Circadian rhythms, orientation, migration, foraging, anti-predator behaviors, mate-detection, and reproduction have all been shown to rely on light conditions and thus these butterfly behaviors are likely to change and perhaps reduce fitness under unnatural light conditions. Future work must delve into the specific mechanisms underlying the ecological impacts of unnatural lighting on butterflies, as this can then inform management and conservation efforts to reduce unnatural lighting in areas and species of concern. However, even before we build this knowledge base, we can mitigate the ecological consequences of unnatural light conditions on butterflies through maintaining mature forests, and reducing the use of anthropogenic lighting at night.

## Figures and Tables

**Table 1:** Mechanisms and biological functions. The four mechanisms with the five butterfly behaviors are listed here with the most pertinent sources. Blank boxes represent biological functions that are likely not affected by the specific mechanism.

Mechanism	Attraction and Orientation	Foraging	Phenology and Circadian Rhythms	Predation	Reproduction
Masking	The built environment could mask polarized light cues that Monarch butterflies use for migration [37,54,55].	Butterflies rely upon visual cues to identify nectar resources and hostplants. Altering light environments will change these visual cues as they rely upon ambient illumination [73,74,75].	Butterflies rely upon the natural light regimes of their habitats for the timing of daily and seasonal activity patterns. Through habitat destruction and anthropogenic lighting, these regimes are masked with unnatural light conditions [76,77,78,79].	Butterflies rely on visual defenses such as deimatic, warning, and cryptic coloration. Through altered light environments, these signals are altered and can increase predation risk [80,81,82,83].	Sexual signals have evolved under specific light conditions and unnatural lighting will mask the visual signal between males and females [84,85,86,87,88].
Distraction	Anthropogenic lights attract, and thus distract, butterflies from normal nocturnal behaviors [89,90,91].			As butterflies are distracted and attracted to anthropogenic lighting, they are more vulnerable to predation [92,93,94].	
Misleading	Altering habitat structure through deforestation and anthropogenic lighting at night changes light environments that mislead butterfly orientation [55,84,85,91,95,96]	As with masking, altering the light environment will change the perceived visual cues of nectar sources and hostplants, which could mislead butterflies into attempting to forage upon the wrong species of plant [73,74,75].	Butterflies use day length as an environmental cue for timing of pupation, eclosion, migration, and diapause. Anthropogenic lighting is increasing day length, which is likely misleading butterflies on when to pupate, eclose, migrate, and begin diapause [76,77,78,79,97,98,99].	Butterflies rely on light environments as a cue for correct habitat and unnatural light environments could mislead butterflies into occupying habitats where survival is decreased due to predation [95,100]. Also, butterflies use natural light regimes for development and when butterflies increase developmental rate due to heightened light levels, predation increases [101].	Butterflies rely on visual cues for courtship and mate detection. Through unnatural lighting and the built environment, both color and polarized light signals could become misleading and butterflies may be courting inappropriate objects [37,52,53].
Temporal Niche		Anthropogenic lighting at night is likely to extend the butterfly activity into dawn and dusk and thus butterflies could be feeding earlier and later in the day [102,103,104,105].		Both butterfly and predator daily temporal patterns are increased by anthropogenic lighting. This increased behavior by butterflies makes them more vulnerable to novel predators (e.g., bats) and their natural predators are also able to hunt earlier and later in the day, increasing predation [106,107]	Butterflies have genital photoreceptors that enable copulation and thus anthropogenic lighting could increase the available time that butterflies are able to copulate [58,59,61,108].
